# Supplementary material for: Atomically unveiling the structure-activity relationship of biomacromolecule-metal-organic frameworks symbiotic crystal
Source: Nat Commun. 2022 Feb 17;13:951. doi: 10.1038/s41467-022-28615-y (PMC8854593; doi:10.1038/s41467-022-28615-y)
Supplement: Supplementary file 1 — Supplementary Information [file 41467_2022_28615_MOESM1_ESM.pdf]

Supplementary information

Atomically unveiling the structure-activity relationship of biomacromolecule-metal-organic frameworks symbiotic crystal

Linjing Tong[1], Siming Huang[2], Yujian Shen[1], Suya Liu[4], Xiaomin Ma[3], Fang Zhu[1], Guosheng Chen[1]*, Gangfeng Ouyang[1]

[1]MOE Key Laboratory of Bioinorganic and Synthetic Chemistry, School of Chemistry, Sun Yat-sen University, Guangzhou 510275, China

[2]School of Pharmaceutical Sciences, Guangzhou Medical University, Guangzhou 511436, China

[3]Cryo-EM Center, Southern University of Science and Technology, Shenzhen, 518055, China

[4]Shanghai Nanoport, Thermo Fisher Scientific, Jinke Road. Pudong District, Shanghai, 200120, China

*Corresponding authors, E-mail: chengsh39@mail.sysu.edu.cn (G. Chen)

Supplementary Table

Supplementary Table 1. The parameters of BZIF-8 crystallized in different pathways

The enzyme dosage used (mg)	Encapsulating content[a] in BZIF-8-B (wt %)	Yields of BZIF-8-B (mg)	Enzymes content in BZIF-8-B (mg)	Encapsulating content[a] in BZIF-8-S (wt %)	Yields of BZIF-8-S (mg)	Enzymes content in BZIF-8-S (mg)
1.0	8.6	11.4	0.98	2.5	34.6	0.87
2.0	16.4	12.0	1.97	5.7	34.5	1.97
4.0	29.2	13.5	3.94	10.7	36.9	3.95
6.0	38.2	15.5	5.92	11.2	38.2	4.28
8.0	48.8	16.2	7.9	12.6	44.7	5.63

[a]The encapsulating content was evaluated by examining the concentration differences of enzyme in the supernatant before and after encapsulation by the standard Bradford assay.

Supplementary Figures

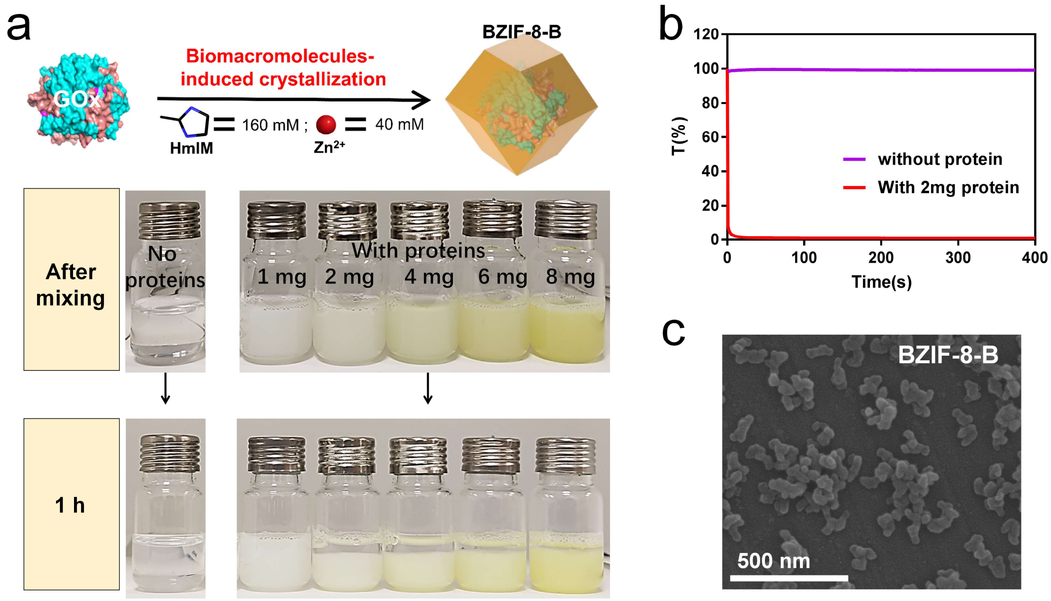

Supplementary Fig. 1. Biomacromolecules-induced crystallization. (a)The photographs recorded the biomacromolecules-induced crystallization system after mixing proteins and ZIF-8 precursor instantaneously. (b) The time-dependent transmittance of the crystallization system with and without proteins. It was observed that the crystallization rate of BZIF-8 was significantly improved when adding the proteins, and it was unable to crystallize without the proteins. (c) SEM images of BZIF-8-B.

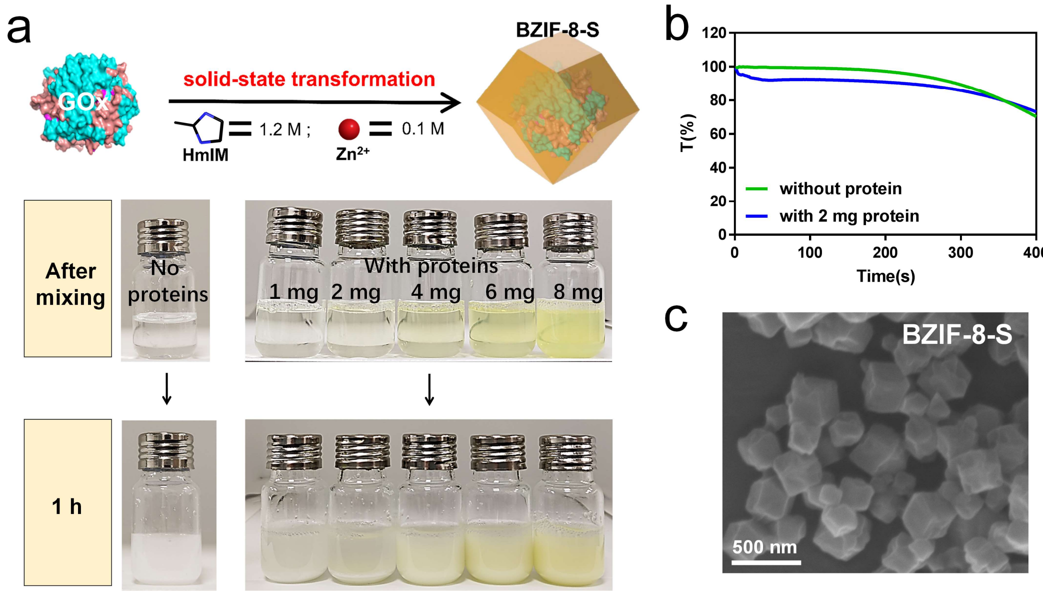

Supplementary Fig. 2. Solid-state transformation crystallization. (a)The photographs recorded the solid-state transformation crystallization system after mixing proteins and ZIF-8 precursor instantaneously. (b) The time-dependent transmittance of the crystallization system with and without proteins. It was observed that the both of the crystallization rates of BZIF-8 were slow, and the crystallization rate was not affected by the proteins. (c) SEM images of BZIF-8-S.

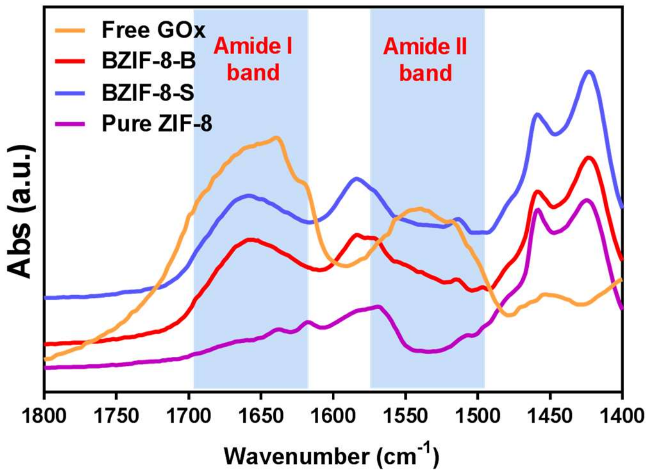

Supplementary Fig. 3. FT-IR spectra. FT-IR spectra of pure ZIF-8, BZIF-8-S, BZIF-8-B and free GOx.

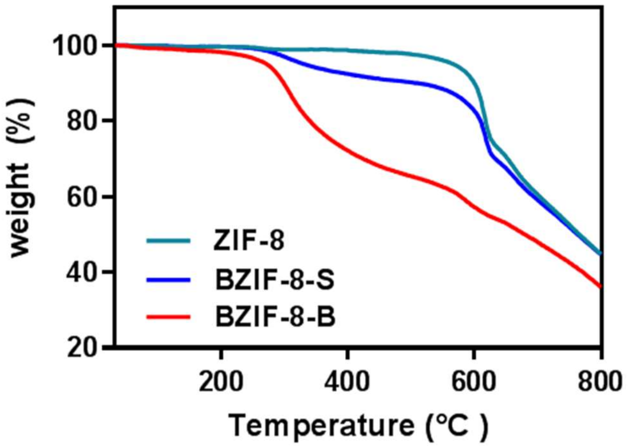

Supplementary Fig. 4. TGA analysis. TGA curves of pure ZIF-8, BZIF-8-S and BZIF-8-B. The weight loss between ca. 250-600 °C was caused by the pyrolysis of the incorporated proteins.

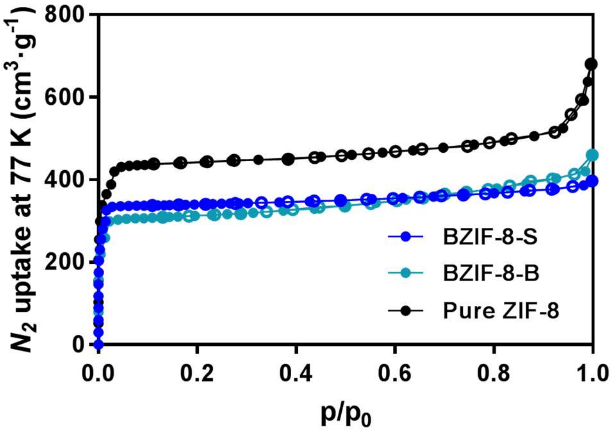

Supplementary Fig. 5. N_2 adsorption/desorption isotherms. N_2 adsorption/desorption isotherms of pure ZIF-8, BZIF-8-S and BZIF-8-B. The N_2 adsorption amounts of BZIF-8-B and iBZIF-8-S were smaller that the than that of the pure ZIF-8. The decreased N_2 adsorption amounts were due to the incorporation of the proteins.

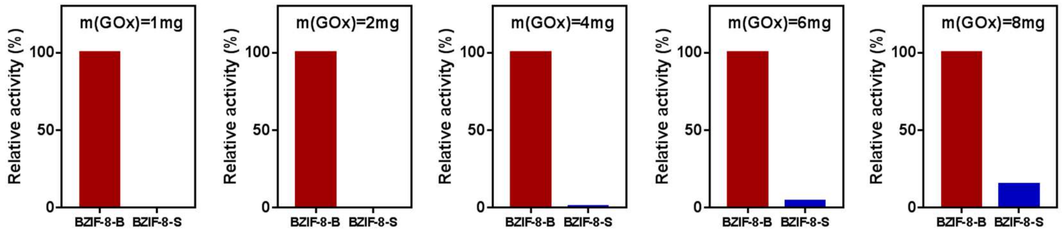

Supplementary Fig. 6. Bioactivities assay between BZIF-8-S and BZIF-8-B. Comparison of relative bioactivities of BZIF-8-S and BZIF-8-B when crystallization using different GOx dosage. The GOx dosage in each trial was kept at 6 μg.

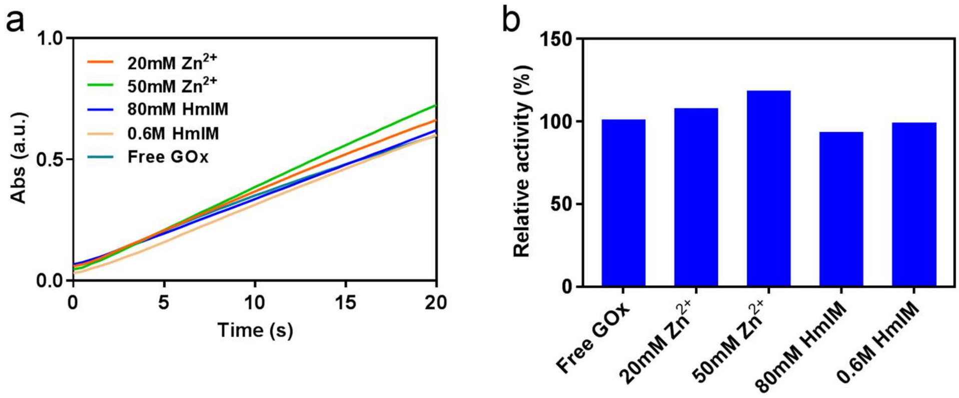

Supplementary Fig. 7. The effect of different treatments on enzyme's activity. Monitoring of the catalytic kinetic curves of free GOx under different condition treatments for 1h. (b) The calculated relative activities based on the initial catalytic rate from (a)

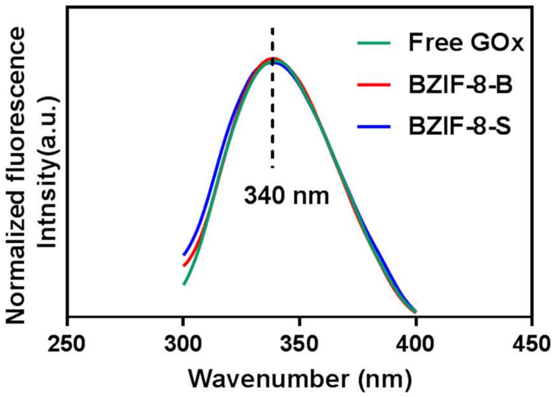

Supplementary Fig. 8. Structural profile by fluorescence spectroscopy. The fluorescence spectroscopy profiles of free GOx, and the GOx encapsulated in BZIF-8-B and BZIF-8-S, respectively. Both the free GOx and encapsulated GOx showed a λ_{max} at 340 nm, indicating that the encapsulated proteins maintained its native structure.

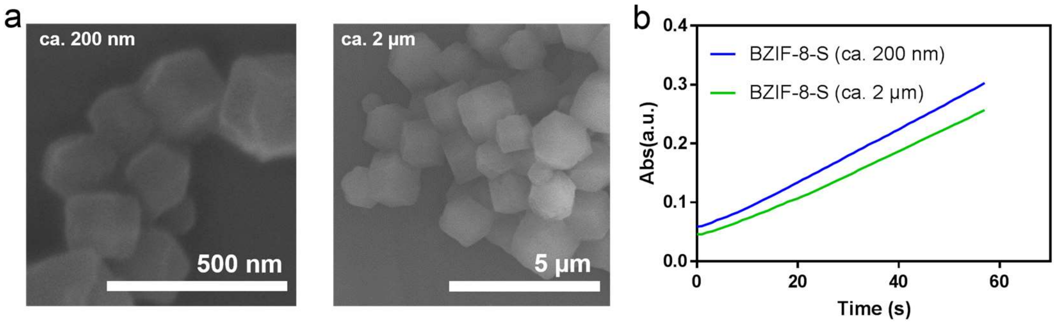

.

Supplementary Fig. 9. The effect of crystal size on the activity of encapsulated enzyme. The SEM images (a) and the bioactivity (b) of prepared BZIF-8-S with ca. 200 nm and 2 μm. The enzymes (GOx) dosage for the activity test in different material were kept the same (14 μg).

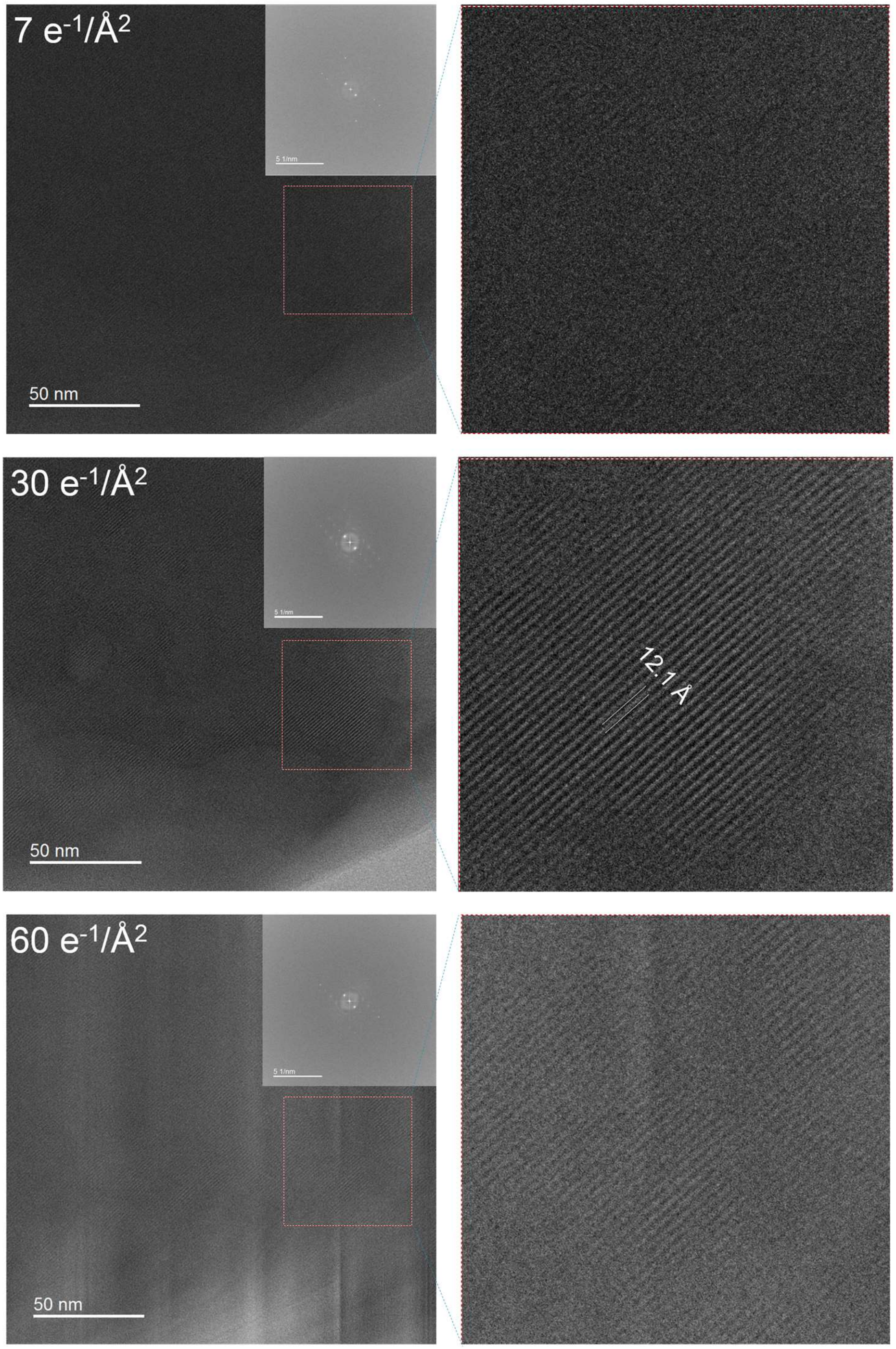

Supplementary Fig. 10. The diffraction resolutions under different electron doses. Cryo-EM images and corresponding diffraction resolution of BZIF-8-S exposed to cumulative electron doses of 7 $e^-/Å^2$, 30 $e^-/Å^2$ and 60 $e^-/Å^2$, respectively.

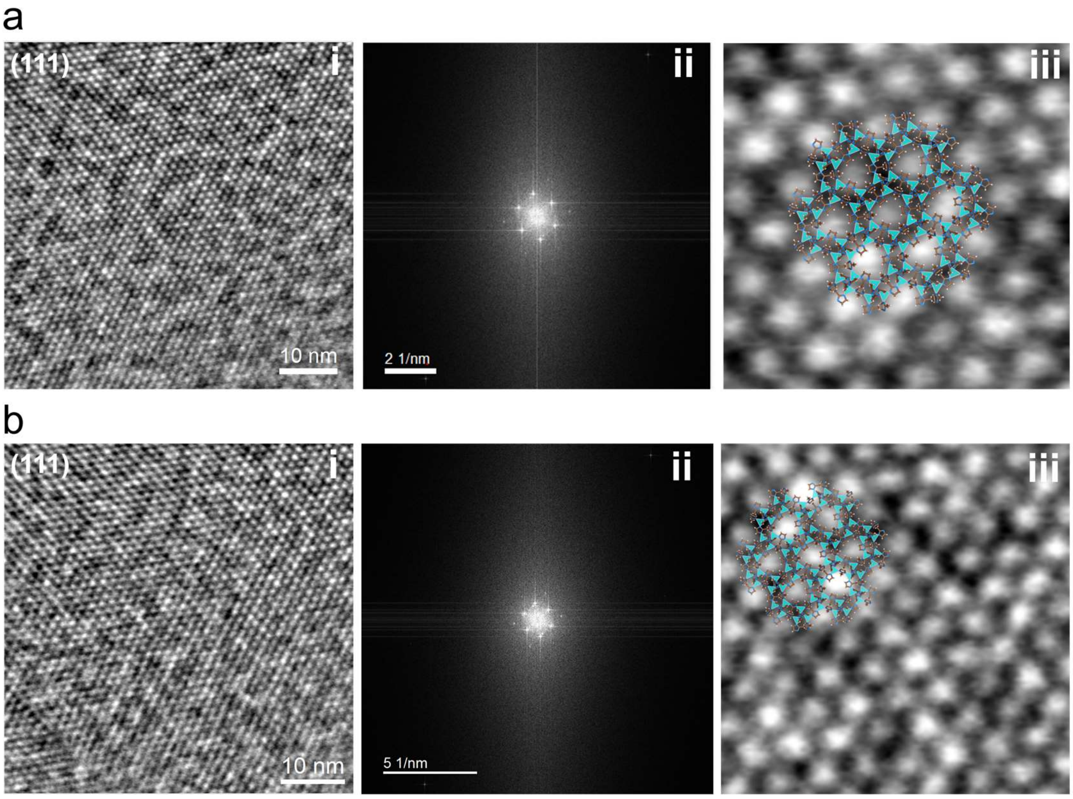

Supplementary Fig. 11. High-resolution structure of BZIF-8-S viewed from the [111] projection. (a) The iDPC-STEM image (i), corresponding FFT pattern (ii) and magnified image (iii) of BZIF-8-S in one randomly selected BZIF-8-S particle, viewing from the [111] projection. (b) The iDPC-STEM image (i), corresponding FFT pattern (ii) and magnified image (iii) of BZIF-8-S in another randomly selected BZIF-8-S particle, viewing from the [111] projection.

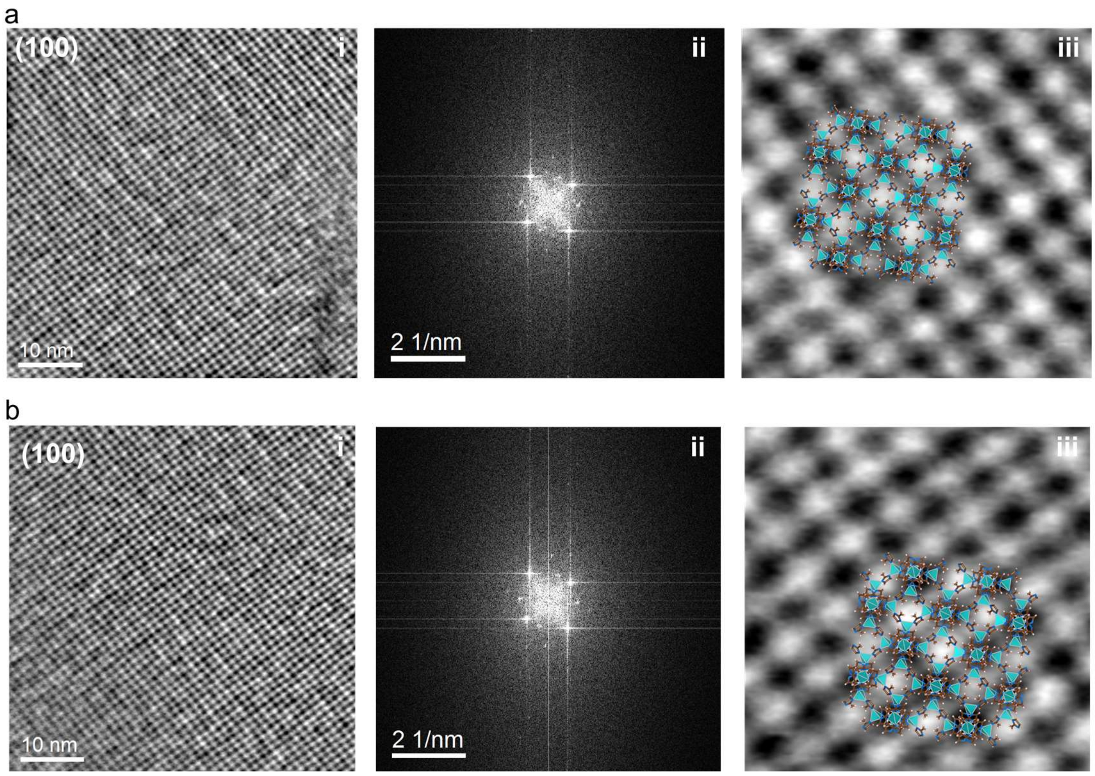

Supplementary Fig. 12. High-resolution structure of BZIF-8-S viewed from the [100] projection. (a) The iDPC-STEM image (i), corresponding FFT pattern (ii) and magnified image (iii) of BZIF-8-S in one randomly selected BZIF-8-S particle, viewing from the [100] projection. (b) The iDPC-STEM image (i), corresponding FFT pattern (ii) and magnified image (iii) of BZIF-8-S in another randomly selected BZIF-8-S particle, viewing from the [100] projection.

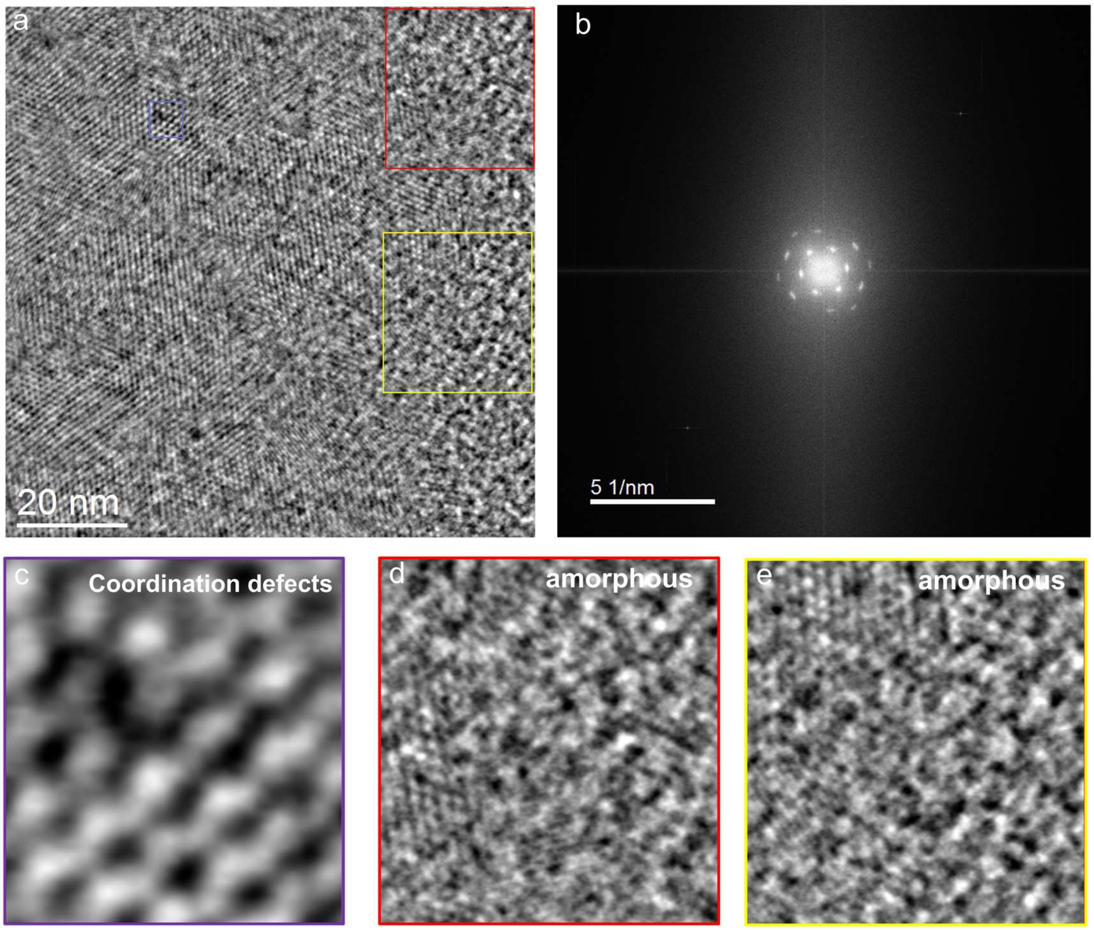

Supplementary Fig. 13. High-resolution structure of randomly selected BZIF-8-B. The iDPC-STEM image of BZIF-8-B (a) and the corresponding FFT pattern (b) in one randomly selected BZIF-8-B particle. (c) Magnified image of purple boxed region from (a). (d) Magnified image of red boxed region from (a). (e) Magnified image of yellow boxed region from (a).

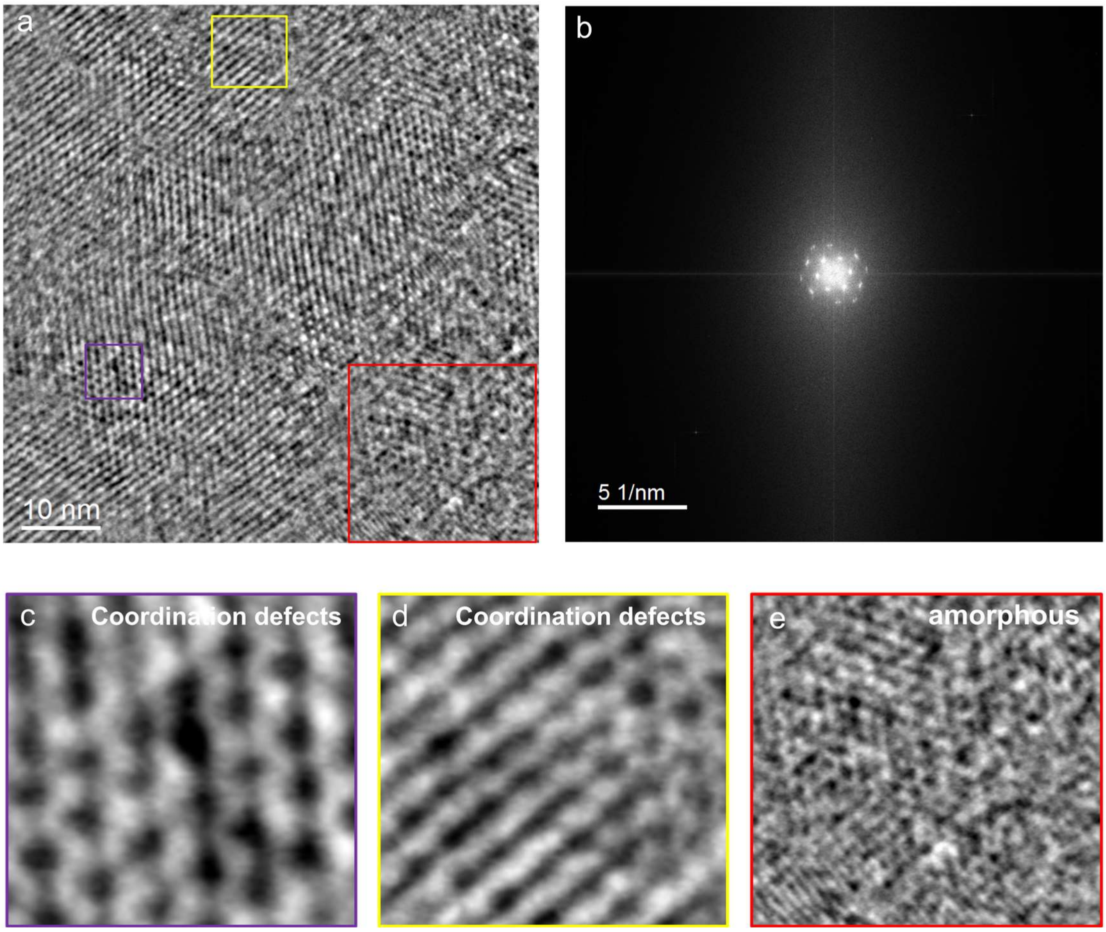

Supplementary Fig. 14. High-resolution structure of randomly selected BZIF-8-B. The iDPC-STEM image of BZIF-8-B (a) and the corresponding FFT pattern (b) in the second randomly selected BZIF-8-B particle. (c) Magnified image of purple boxed region from (a). (d) Magnified image of red boxed region from (a). (e) Magnified image of yellow boxed region from (a).

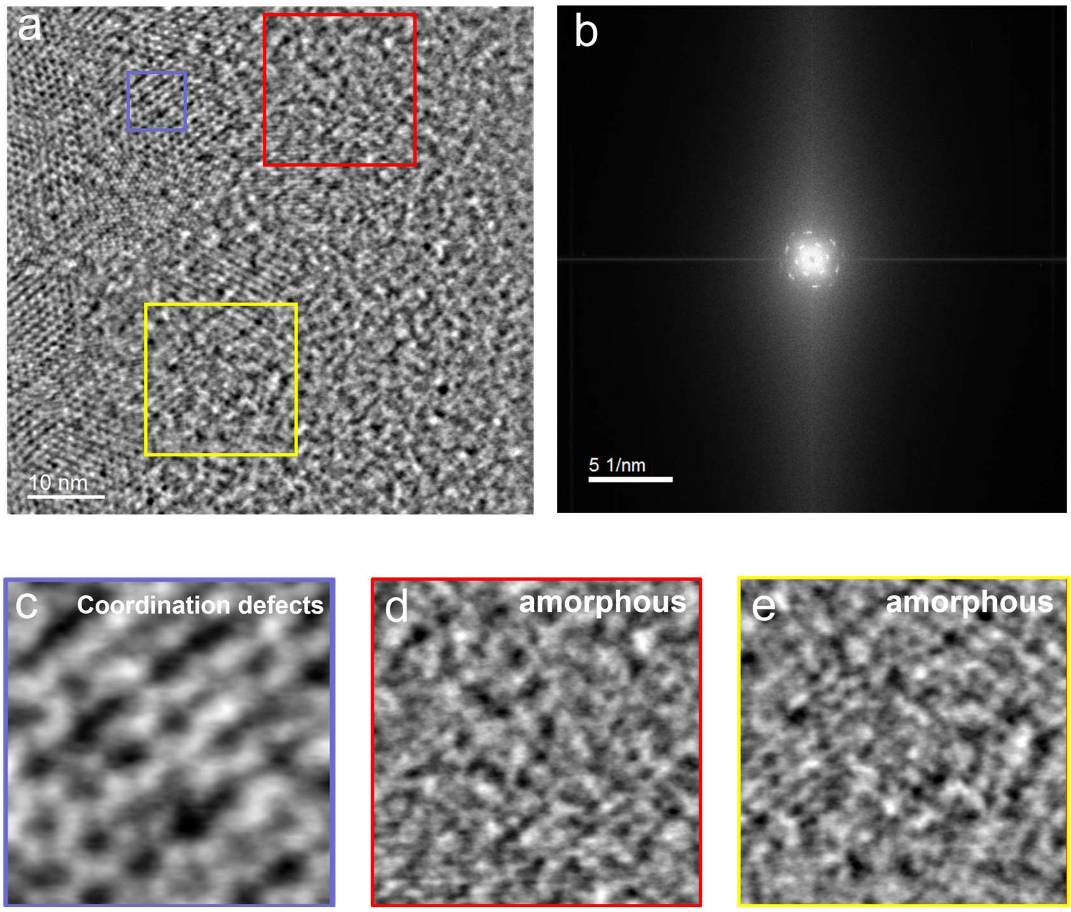

Supplementary Fig. 15. High-resolution structure of randomly selected BZIF-8-B. The iDPC-STEM image of BZIF-8-B (a) and the corresponding FFT pattern (b) in the third randomly selected BZIF-8-B particle. (c) Magnified image of purple boxed region from (a). (d) Magnified image of red boxed region from (a). (e) Magnified image of yellow boxed region from (a).

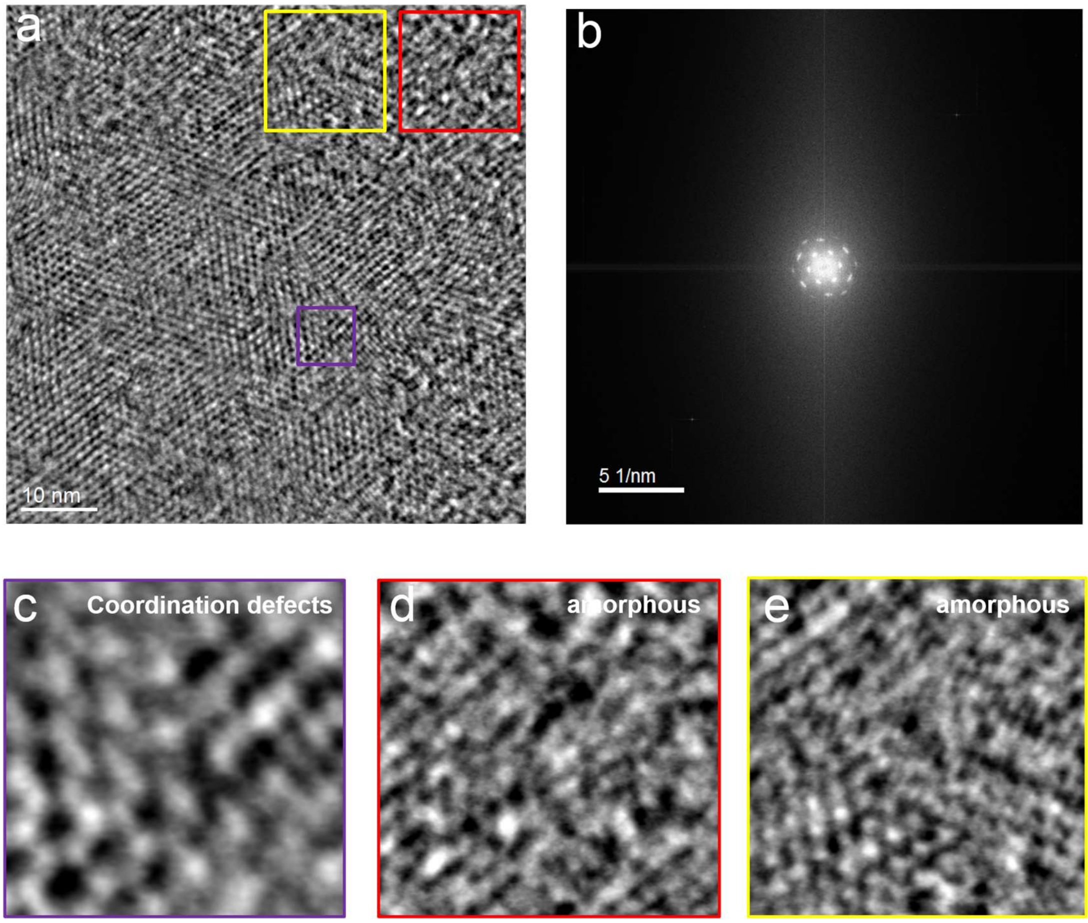

Supplementary Fig. 16. High-resolution structure of randomly selected BZIF-8-B. The iDPC-STEM image of BZIF-8-B (a) and the corresponding FFT pattern (b) in the fourth randomly selected BZIF-8-B particle. (c) Magnified image of purple boxed region from (a). (d) Magnified image of red boxed region from (a). (e) Magnified image of yellow boxed region from (a).

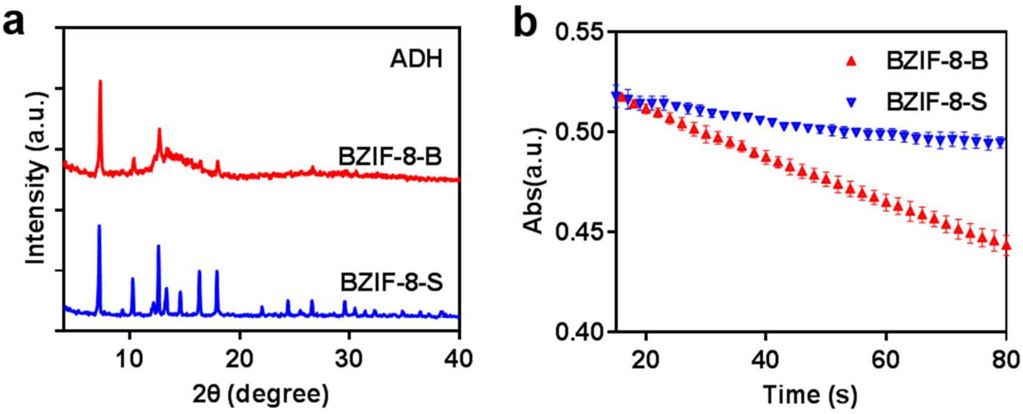

Supplementary Fig. 17. ADH@BZIF-8 synthesis and activity characterization. (a) PXRD patterns of BZIF-8-B and BZIF-8-S in which ADH were encapsulated within ZIF-8. 8 mg ADH was used for the BZIF-8 crystallization. (b) Monitoring of the catalytic activities of ADH of BZIF-8-B and BZIF-8-S. Error bars = Standard Deviation (n=3).

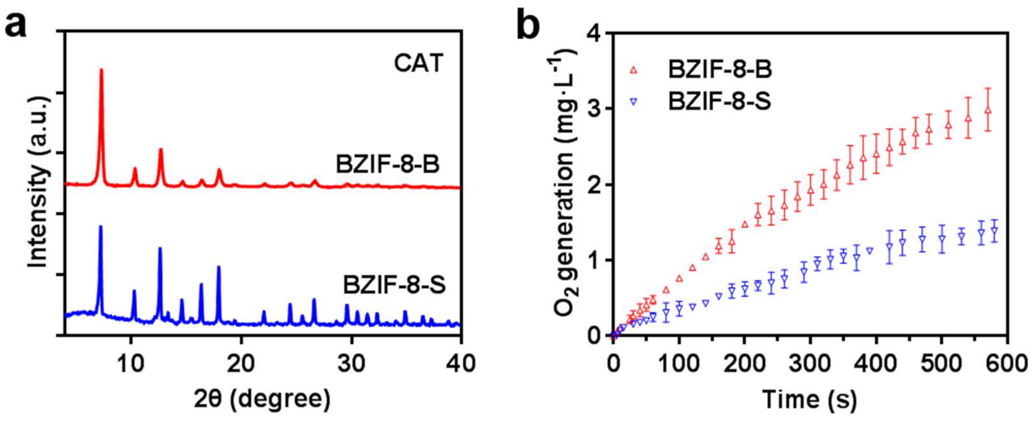

Supplementary Fig. 18. CAT@BZIF-8 synthesis and activity characterization. (a) PXRD patterns of BZIF-8-B and BZIF-8-S in which CAT were encapsulated within ZIF-8. 8 mg CAT was used for the BZIF-8 crystallization. (b) Monitoring of the catalytic activities of CAT of BZIF-8-B and BZIF-8-S. Error bars = Standard Deviation (n=3).

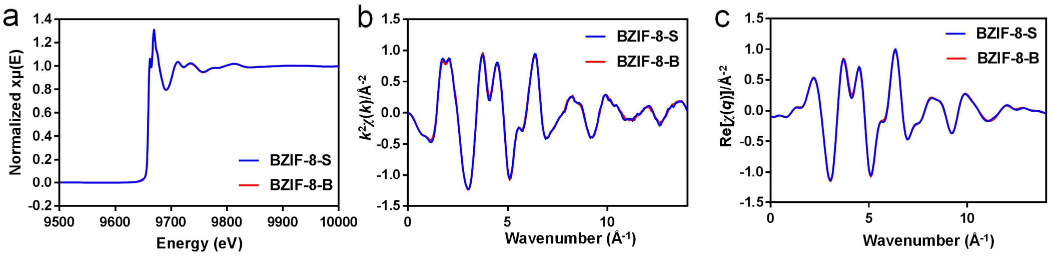

Supplementary Fig. 19. XAFS analysis. Normalized XAFS spectra (a), EXAFS *k*-space spectra (b), and EXAFS *q*-space spectra (c) of BZIF-8-S and BZIF-8-B at Zn K-edge.

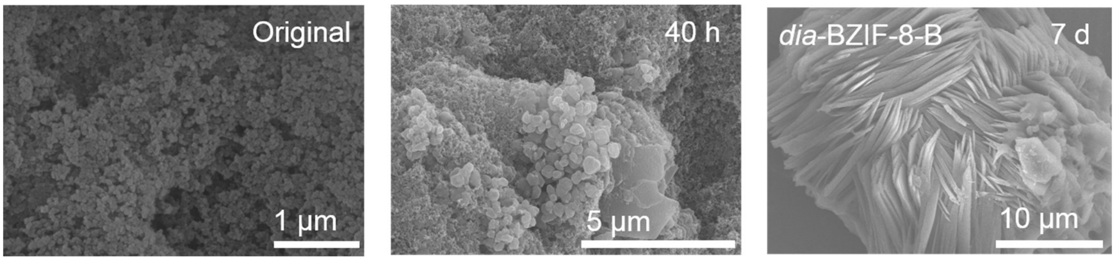

Supplementary Fig. 20. Morphology evolution. SEM images of the fresh-prepared BZIF-8-B crystals, and the crystals when prolonging the crystallization time to 40 h and 7d.

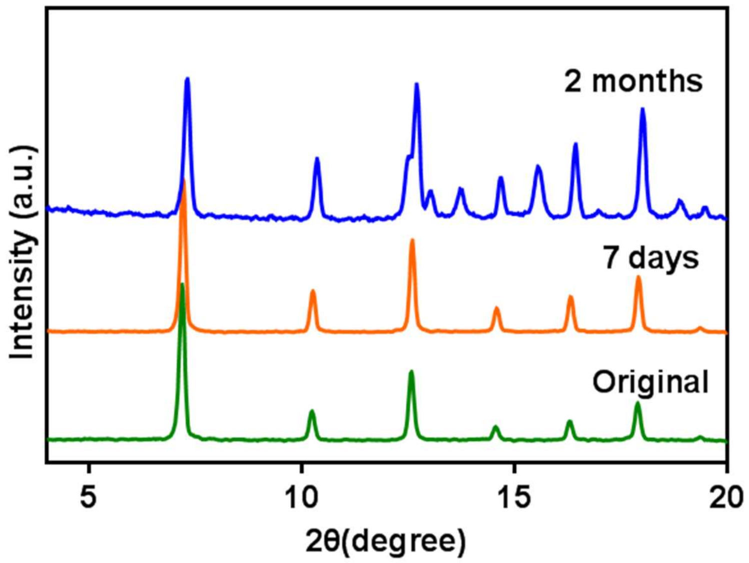

Supplementary Fig. 21. PXRD profile. PXRD patterns of the fresh-prepared BZIF-8-S crystals, and the crystals when prolonging the crystallization time to 7 d and 2 months.

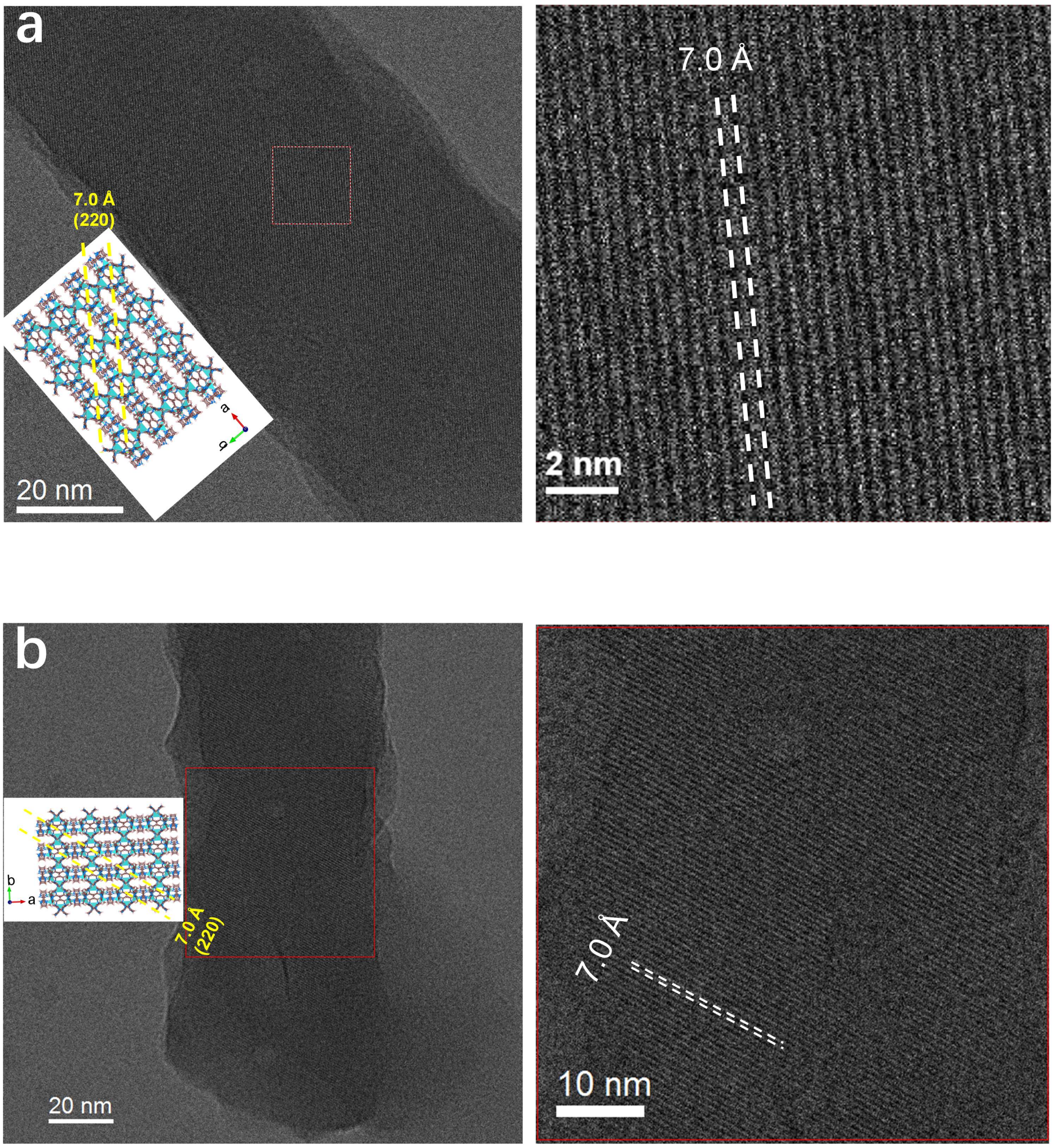

Supplementary Fig. 22. Cryo-EM structure of *dia*-BZIF-8-B crystals. The cryo-EM image showed the highly crystalline structure of two randomly selected *dia*-BZIF-8-B crystals (a) and (b).

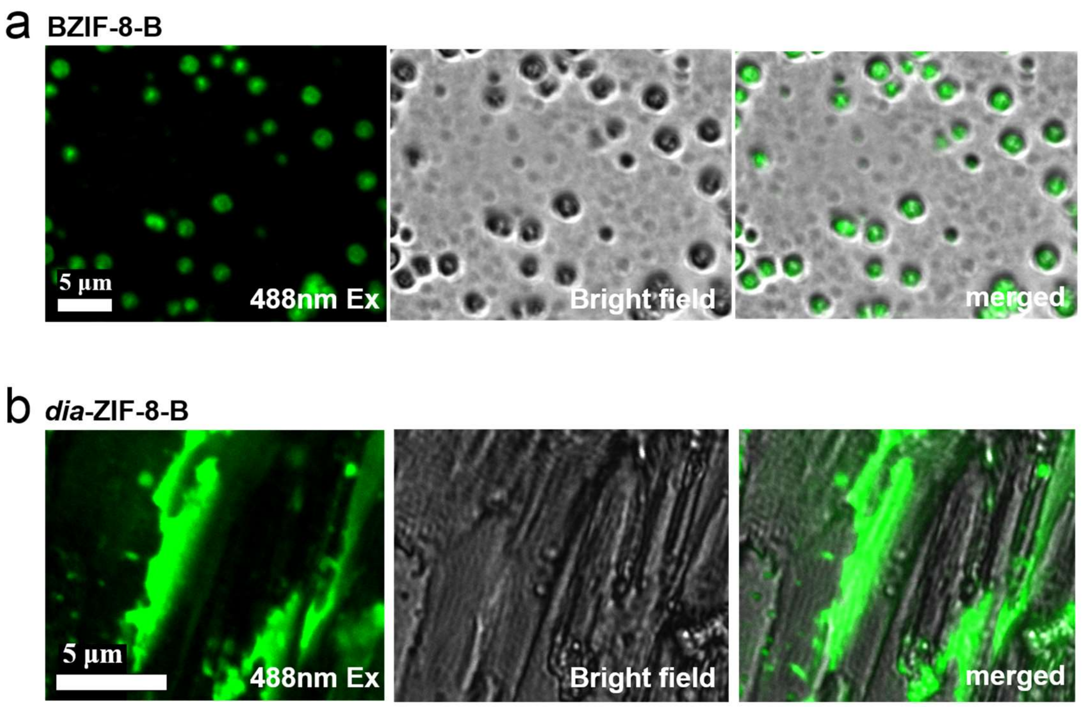

Supplementary Fig. 23. The enzymes spatial distribution. CLSM presented the spatial distribution of GOx (green fluorescence) within the original BZIF-8-B and the phase-transformed *dia*-BZIF-8-B.

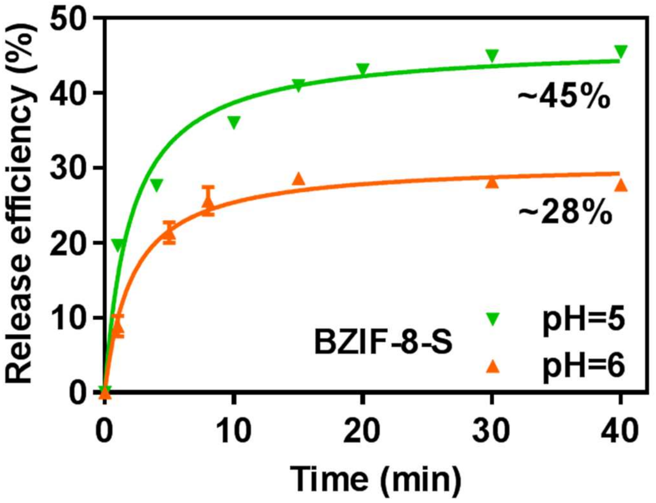

Supplementary Fig. 24. The pH-dependent release. The protein release efficiency of BZIF-8-S at pH 5 and 6 PBS. Error bars = Standard Deviation (n=3).
